# “*Candidatus* Galacturonibacter soehngenii” Shows Acetogenic Catabolism of Galacturonic Acid but Lacks a Canonical Carbon Monoxide Dehydrogenase/Acetyl-CoA Synthase Complex

**DOI:** 10.3389/fmicb.2020.00063

**Published:** 2020-01-29

**Authors:** Laura C. Valk, Martijn Diender, Gerben R. Stouten, Jette F. Petersen, Per H. Nielsen, Morten S. Dueholm, Jack T. Pronk, Mark C. M. van Loosdrecht

**Affiliations:** ^1^Department of Biotechnology, Delft University of Technology, Delft, Netherlands; ^2^Laboratory of Microbiology, Wageningen University and Research, Wageningen, Netherlands; ^3^Department of Chemistry and Bioscience, Centre for Microbial Communities, Aalborg University, Aalborg, Denmark

**Keywords:** acetogenesis, ^13^C-labeling, meta-transcriptomics, chemostat enrichment culture, Wood-Ljungdahl pathway

## Abstract

Acetogens have the ability to fixate carbon during fermentation by employing the Wood-Ljungdahl pathway (WLP), which is highly conserved across Bacteria and Archaea. In a previous study, product stoichometries in galacturonate-limited, anaerobic enrichment cultures of “*Candidatus* Galacturonibacter soehngenii,” from a novel genus within the *Lachnospiraceae*, suggested the simultaneous operation of a modified Entner-Doudoroff pathway for galacturonate fermentation and a WLP for acetogenesis. However, a draft metagenome-assembled genome (MAG) based on short reads did not reveal homologs of genes encoding a canonical WLP carbon-monoxide-dehydrogenase/acetyl-Coenzyme A synthase (CODH/ACS) complex. In this study, NaH^13^CO_3_ fed to chemostat-grown, galacturonate-limited enrichment cultures of “*Ca.* G. soehngenii” was shown to be incorporated into acetate. Preferential labeling of the carboxyl group of acetate was consistent with acetogenesis via a WLP in which the methyl group of acetate was predominately derived from formate. This interpretation was further supported by high transcript levels of a putative pyruvate-formate lyase gene and very low transcript levels of a candidate gene for formate dehydrogenase. Reassembly of the “*Ca.* G. soehngenii” MAG with support from long-read nanopore sequencing data produced a single-scaffold MAG, which confirmed the absence of canonical CODH/ACS-complex genes homologs. However, high CO-dehydrogenase activities were measured in cell extracts of “*Ca.* G. soehngenii” enrichment cultures, contradicting the absence of corresponding homologs in the MAG. Based on the highly conserved amino-acid motif associated with anaerobic Ni-CO dehydrogenase proteins, a novel candidate was identified which could be responsible for the observed activities. These results demonstrate operation of an acetogenic pathway, most probably as a yet unresolved variant of the Wood-Ljungdahl pathway, in anaerobic, galacturonate-limited cultures of “*Ca.* G. soehngenii.”

## Introduction

Over the course of multiple decades, seven carbon-fixing pathways capable of supporting autotrophic growth have been identified and intensively studied; the Calvin-Benson-Bassham (CCB) reductive pentose-phosphate cycle, the reductive citric-acid cycle (Arnon-Buchanan (AB) cycle), the hydroxypropionate (Fuchs-Holo) bi-cycle, the 3-hydroxypropionate/4-hydroxybutyrate cycle, dicarboxylate/hydroxybutyrate cycle, the reductive acetyl-CoA (Wood-Ljungdahl) pathway and the reductive glycine pathway (Berg, 2011; Fuchs, 2011; Figueroa et al., 2018). The first five pathways are primarily used for carbon fixation and the reductive glycine pathway for recycling of electron carriers. Only the Wood-Ljungdahl pathway (WLP) also acts as a primary pathway for energy conservation in anaerobes (Fuchs, 2011; Bar-Even et al., 2012b; Schuchmann and Müller, 2014).

The WLP is highly conserved across Archaea and Bacteria, with only two known variations, one found predominantly in methanogenic archaea and one in acetogenic bacteria. The first has formyl-methanofuran rather than formate as first intermediate, and uses ATP-independent formyl-MFR:tetrahydromethanopterin formyltransferase instead of ATP-consuming formyl-tetrahydrofolate ligase (consuming an ATP). Moreover, methanogens use methanofuran (MFR), tetrahydromethanopterin and coenzyme-F_420_ as cofactors while acetogens rely on NAD(P)H, tetrahydrofolate (THF) and ferredoxin (Fd) (Fuchs, 2011; Adam et al., 2018). Reduction of CO_2_ to acetate via the WLP requires 8 electrons (Equation 1, Ragsdale and Pierce, 2008; Schuchmann and Müller, 2014).

$$2\,C\,O_{2}+8\,H^{+}+8\,e^{-}+n\,A\,D\,P+n\,P_{i}\to$$

(1) $$C\,H_{3}\,C\,O\,O\,H+n\,A\,T\,P+\left(2+n\right)\,H_{2}\,O$$

The WLP consists of two branches. In acetogens, the WLP methyl branch reduces CO_2_ to a methyl group by first reducing CO_2_ to formate via formate dehydrogenase (*fdhA*; EC 1.17.1.9), after which formate is bound to tetrahydrofolate (THF) by formate-tetrahydrofolate ligase (*fhs*, EC 6.3.4.3). Formyl-THF is then further reduced to methenyl-THF, methylene-THF and lastly to methyl-THF by formyl-THF cyclohydrolase and methylene-THF dehydrogenase (*folD*; EC 3.5.4.9 and EC 1.5.1.5) and methylene-THF reductase (*metF*, EC 1.5.1.20), respectively (Ragsdale, 2008; Ragsdale and Pierce, 2008). A methyl transferase then transfers the methyl group from THF to a corrinoid iron–sulfur protein (*acsE*, EC 2.3.1.258), which is a subunit of the carbon monoxide (CO) dehydrogenase/acetyl-CoA synthase complex. The carbonyl branch of the WLP reduces CO_2_ to CO in a reaction catalyzed by another subunit of the canonical WLP, the CO dehydrogenase/acetyl-CoA synthase complex (CODH/ACS, EC 2.3.1.169). Alternatively, CO can be formed by a separate CO dehydrogenase (CODH, EC 1.2.7.4) (Ragsdale and Kumar, 1996; Doukov et al., 2002; Jeoung and Dobbek, 2011). The CODH/ACS complex then links the two WLP branches by coupling the CO- and CH_3_-groups with CoA, yielding acetyl-CoA (Menon and Ragsdale, 1996a; Ragsdale and Kumar, 1996; Ragsdale, 2008). The high degree of conservation of WLP genes and their genomic co-localization suggests that their evolution involved interspecies gene transfer events (Techtmann et al., 2012; Adam et al., 2018). However, two recent studies suggested carbon fixation occurred in the absence of a full complement of structural genes for canonical WLP enzymes (Figueroa et al., 2018; Valk et al., 2018). These observations suggest that variants of the canonical WLP may still await discovery.

In a recent study on D-galacturonate-limited, anaerobic enrichment cultures, we identified the dominant bacterium as a species from a novel genus within the *Lachnospiraceae*, for which we proposed the name “*Candidatus* Galacturonibacter soehngenii.” The *Lachnospiraceae* family is part of the phylum Firmicutes, which includes several genera that harbor acetogens (Drake et al., 2008; Ragsdale and Pierce, 2008; Schuchmann and Muller, 2013; Valk et al., 2018). Fermentation product stoichiometries of the enrichment cultures were consistent with an acetogenic dissimilation of galacturonate. The overall stoichiometry is shown in Equation (2) (Valk et al., 2018).

(2) $$1\,C_{6}\,H_{10}\,O_{7}\to 2.5\,C_{2}\,H_{4}\,O_{2}+1\,C\,O_{2}$$

Metagenome analysis of the enrichment culture revealed homologs of most structural genes for WLP enzymes, but no homologs were found for genes encoding subunits of the canonical CODH/ACS complex (EC 2.3.1.169) (Valk et al., 2018).

The goal of the present study was to further investigate the presence of a possible alternative configuration of the WLP in “*Ca.* G. soehngenii.” To analyze *in vivo* activity of the WLP, D-galacturonate-limited enrichment cultures were co-fed with ^13^C-labeled bicarbonate, followed by analysis of ^13^C in the methyl and carboxyl groups of acetate. To investigate whether canonical WLP genes might have been overlooked in the initial metagenomics analysis, a fully closed metagenome-assembled genome (MAG) sequence of “*Ca.* G. soehngenii” was constructed using long-read nanopore sequencing, and meta-transcriptome analysis was performed to analyze the expression levels of genes of interest. Additionally, CO dehydrogenase activity was analyzed in cell extracts.

## Materials and Methods

### Reactor Setup and Operation

Chemostat cultures were grown in 1.2 L laboratory bioreactors (Applikon, Delft, The Netherlands), which were stirred at 300 rpm and kept at 30°C. Anaerobic conditions were maintained by flushing the headspace with nitrogen gas, at a flow rate of 120 mL min^–1^. Culture pH was controlled at 8 ± 0.1 by automatic titration (ADI 1030 Biocontroller, Applikon, Delft, The Netherlands) of 1 M NaOH. The dilution rate was 0.09 ± 0.01 h^–1^ and the working volume of 0.5 L was kept constant by peristaltic effluent pumps (Masterflex, Cole-Parmer, Vernon Hills, IL, United States) coupled to electrical level sensors. Bioreactors were inoculated (10% v/v) with 50 mL samples of D-galacturonate-limited, anaerobic chemostat enrichment cultures (Valk et al., 2018), stored in 30% v/v glycerol at –20°C. Cultures were run in continuous mode and after at least 6 days (18 generations) stable product composition and biomass concentration were established. System stability was assessed by online monitoring of CO_2_ production and offline monitoring of fermentation products and optical density. When measurements varied by less than 10% over multiple volume changes, without a clear upward or downward trend, samples were taken during subsequent cycles.

### Medium

The cultivation medium contained (g L^–1^): D-galacturonate 4.3; NH_4_Cl 1.34; KH_2_PO_4_ 0.78; Na_2_SO_4_.10H_2_O 0.130; MgCl_2_.6H_2_O 0.120; FeSO_4_.7H_2_O 0.0031; CaCl_2_ 0.0006; H_3_BO_4_ 0.0001; Na_2_MoO_4_. 2H_2_O 0.0001; ZnSO_4_.7H2O 0.0032; CoCl_2_.H_2_O 0.0006; CuCl_2_.2H_2_O 0.0022; MnCl_2_.4H_2_O 0.0025; NiCl_2_.6H_2_O 0.0005; EDTA 0.10. Nineteen liter of mineral solution (mineral concentration adjusted to the final volume, 20 L) was autoclaved for 20 min at 121°C after which 1 L (86 g L^–1^) D-galacturonate solution was filter sterilized (0.2 μm Mediakap Plus, Spectrum Laboratories, Rancho Dominguez, CA, United States) into the media. 1.5 mL Pluronic PE 6100 antifoam (BASF, Ludwigshafen, Germany) was added per 20 L of mineral solution to avoid excessive foaming.

### Analysis of Substrate and Extracellular Metabolite Concentrations

To determine substrate and extracellular metabolite concentration, reactor sample supernatant was obtained by centrifugation of culture samples (Heraeus Pico Microfuge, Thermo Fisher Scientific, Waltham, MA, United States). Concentrations of D-galacturonate and extracellular metabolites were analyzed with an Agilent 1100 Affinity HPLC (Agilent Technologies, Amstelveen, The Netherlands) equipped with an Aminex HPX-87H ion-exchange column (BioRad, Hercules, CA, United States) operated at 60°C with a mobile phase of 5 mM H_2_SO_4_ and a flow rate of 0.6 mL min^–1^. CO_2_ and H_2_ concentrations in the bioreactor exhaust gas were measured using a Prima BT Bench Top mass spectrometer (Thermo Fisher Scientific, Waltham, MA, United States) after the gas was cooled by a condenser (4°C).

### Biomass Dry Weight

Twenty milliliter of culture broth samples were filtered over pre-dried and pre-weighed membrane filters (0.2 μm Supor-200, Pall Corporation, New York, NY, United States), which were then washed with demineralized water, dried in a microwave oven (Robert Bosch GmbH, Gerlingen, Germany) for 20 min at 360 W and reweighed. Carbon and electron balances were constructed based on the number of carbon atoms and electrons per mole, while biomass composition was assumed to be CH_1__.__8_O_0__.__5_N_0__.__2_ (Roels, 1983).

### Quantitative Fluorescent *in situ* Hybridization (qFISH) Analysis

Fluorescent *in situ* hybridization was performed as described previously (Daims et al., 2005), using a hybridization buffer containing 35% (v/v) formamide. Probes were synthesized and 5′ labeled with either 5(6)-carboxyfluorescein-N-hydroxysuccinimide ester (FLUOS) or with one of the sulfoindocyanine dyes (Cy3 and Cy5; Thermo Hybaid Interactiva, Ulm, Germany) (Table 1). The general probe EUB338mix, labeled at both 3′ and 5′ ends with Cy5, was used to identify all eubacteria in the sample. Microscopic analysis was performed with a LSM510 Meta laser scanning confocal microscope (Carl Zeiss, Oberkochen, Germany). The qFISH analysis was based on at least 29 fields of view at 6730 × magnification, using DAIME (version 2.1) software (DOME, Vienna, Austria; Daims et al., 2006). The bio-volume fractions of “*Ca.* G. soehngenii” and *Enterobacteriaceae* populations were calculated as the ratio of the area hybridizing with specific probes relative to the total area hybridizing with the universal EUBmix probe set (Amann et al., 1990; Daims et al., 1999).

**TABLE 1:** Oligonucleotide probes used for the quantitative fluorescence *in situ* hybridization analysis.

**Probe**	**Sequence (5′–3′)**	**Specificity**	**References**
EUB338mix	GCWGCCWCCCGTAGGWGT	All bacteria	Daims et al., 1999
ENT	CTCTTTGGTCTTGCGACG	*Enterobacteriaceae*	Kempf et al., 2000
Lac87	GTGGCGATGCAAGTCTGA	*“Ca.* G. soehngenii”	This study

**With W indicating A or T.**

### Labeling Experiment ^13^C-Labeled Sodium Bicarbonate Addition

A 1 M NaH^13^CO_3_ solution was used to replace the regular 1 M NaOH solution as a pH titrant in steady-state D-galacturonate-limited enrichment cultures (pH 7.8 ± 0.1, D = 0.1 h^–1^, T = 30°C). Broth was collected on ice every 2 h for 8 consecutive hours and centrifuged (12,000 × *g*, Heraeus Pico Microfuge, Thermo Fisher Scientific, Waltham, MA, United States) before the supernatant was collected and stored at –20°C until analysis by NMR. CO_2_, H_2_ and ^13^CO_2_ concentrations in the exhaust gas were measured by MS (Prima BT Bench Top MS, Thermo Fisher Scientific, Waltham, MA, United States) after the gas had been cooled by a condenser (4°C).

### Illumina and Nanopore Sequencing, Metagenome Assembly, and Genome Binning DNA

The metagenomic-assembled genome of “*Candidatus* Galacturonibacter soehngenii” described by Valk et al. (2018) was used as template for preparing the metagenome libraries. The DNA extraction, Illumina sequencing, metagenomic assembly and binning process is described in Valk et al. (2018). Long-read genomic DNA sequencing was conducted using 1D nanopore sequencing (Oxford Nanopore Technologies, Oxford, United Kingdom), following the manufacturer’s protocol (LSK-108), omitting the optional DNA shearing and DNA repair steps. The library was loaded on a flow cell (FLO-MIN106) and the MinION Mk1B DNA sequencer (Oxford Nanopore Technologies, Oxford, United Kingdom) was used for sequencing combined with the MinKNOW v. 1.7.3 (Oxford Nanopore Technologies, Oxford, United Kingdom) software with the 48 h sequencing workflow (NC_48h_Sequencing_Run_FLO_MIN106_SQK-LSK108.py). Albacore v. 1.2.1 (Oxford Nanopore Technologies, Oxford, United Kingdom) was used to base-call the sequencing reads.

### Genome Assembly

The assembling of the contigs from the “*Candidatus* Galacturonibacter soehngenii” genome bin into a single scaffold based on the long Nanopore reads was done using SSPACE-LongRead scaffolder v. 1.1 (Boetzer and Pirovano, 2014). GapFiller v. 1.11 (Boetzer and Pirovano, 2012) or by manual read mapping and extension in CLC Genomics Workbench v. 9.5.2 (Qiagen, Hilden, Germany) were used to close gaps in the draft genome with the previously assembled Illumina data. Finally, manual polishing of the complete genome was done to remove SNPs and ensure a high-quality assembly. The meta-genome has been submitted to the sequence read archive (SRA)^1^ with accession number SRR10674409, under the BioProject ID PRJNA566068.

### Genome Annotation and Analysis

The metagenome-assembled genome was uploaded to the automated Microscope platform (Vallenet et al., 2006, 2017). Manual assessment of pathway annotations was assisted by the MicroCyc (Caspi et al., 2008), KEGG (Kyoto Encyclopedia of Genes and Genomes; Kanehisa et al., 2014) and SwissProt alignment (BLASTP version 2.2.28+; Altschul et al., 1997) databases. The predicted proteome of “*Ca.* G. soehngenii” was submitted to InterProScan (version 5.25-64.0), to identify predictive Pfam domains (El-Gebali et al., 2018). The annotated genome sequence of “*Candidatus* Galacturonibacter soehngenii” has been submitted to the European Nucleotide Archive (ENA) under the BioProject ID PRJNA566068.

### Genome-Centric Meta-Transcriptomic Analyses; RNA Extraction and Purification

During pseudo-steady state, broth samples were taken from the enrichment culture, directly frozen in liquid nitrogen and subsequently stored at −80°C. Five hundred microliter samples were thawed on ice, pelleted by centrifugation (21,000 × *g*, 2 min, 4°C) and used for total RNA extraction with the RNeasy PowerMicrobiome Kit (Qiagen, Hilden, Germany), following the manufacturer’s instruction with the addition of phenol:chloroform:isoamy alcohol (25:25:1) and β-mercaptoethanol (10 μL mL^–1^ final concentration). Cell lysis was with a FastPrep-24 bead beater (MP Biomedicals, Fisher Scientific, Hampton, VA, United States, four successive cycles of 40 s at 6.0 m s^–1^, 2 min incubation on ice between cycles). Total RNA extracts were subjected to DNase treatment to remove DNA contaminants by using the DNase Max Kit (Qiagen, Hilden, Germany) and further cleaned up and concentrated with the Agencourt AMpure XP magnetic beads (Beckman Coulter, Brea, CA, United States) before rRNA depletion. Integrity and quality of purified total RNA were assessed on a Tapestation 2200 (Agilent, Santa Clara, CA, United States) with the Agilent RNA screen-tapes (Agilent, Santa Clara, CA, United States) and the concentration was measured using Qubit RNA HS Assay Kit (Thermo Scientific Fisher, Waltham, MA, United States).

### rRNA Depletion, Library Preparation, and Sequencing

Five hundred nanogram of total RNA from each sample was obtained after rRNA was depleted using the Ribo-Zero rRNA Removal (Bacteria) Kit (Illumina, San Diego, CA, United States), with 2 μg total RNA as input. Quality of extracted mRNA was checked with Agilent RNA HS screen-tapes (Agilent, Santa Clara, CA, United States) and RNA concentration was determined with a Qubit RNA HS Assay Kit (Thermo Scientific Fisher, Waltham, MA, United States). The TruSeq Stranded mRNA Sample Preparation Kit (Illumina, San Diego, CA, United States) was used to prepare cDNA sequencing libraries according to the manufacturer’s instruction. Libraries were sequenced on an Illumina HiSeq2500 using the TruSeq PE Cluster Kit v3-cBot-HS and TruSeq SBS kit v.3-HS sequencing kit (1 × 50 bp; Illumina, San Diego, CA, United States). The raw meta-transcriptome reads have been submitted to the sequence read archive (SRA)^1^ with accession number SRR10674118-23, under the BioProject ID PRJNA566068.

### Trimming and Mapping of rRNA Reads

Raw RNA reads in FASTQ format were imported into CLC Genomics Workbench v. 9.5.5 and trimmed for quality, requiring a minimum phred score of 20 and a read length of 45. Reads from each sample were hereafter mapped to CDSs obtained from the MAG of “*Ca.* G. soehngenii” with a minimum similarity of 98% over 80% of the read length. Reads per kilobase of transcript per million mapped reads (RPKM) were calculated based on raw read-counts and the length of each CDS. The meta-transcriptome mapped to the genome of “*Ca*. G. soehngenii” are shown in Supplementary Data Sheet S2.

### Plasmid and Strain Construction

Gene F7O84_RS11645 was codon optimized for expression in *Escherichia coli* with the GeneArt online tool and integrated behind the TEV recognition site of the pET151/D-TOPO expression vector by GeneArt (GeneArt GmbH, Regensburg, Germany). The resulting plasmid was transformed into a chemically competent *E. coli* strain BL21 according to manufacturer’s instructions (NEBuilder HiFi DNA Assembly Master Mix chemical transformation protocol (E2621), New England Biolabs, Ipswich, MA, United States) and named pUD1074. The plasmid sequence of pUD1074 has been deposited at the NCBI GenBank^2^ with the corresponding accession number MN498128.

### Heterologous Expression of the Putative CO Dehydrogenase Candidate

All *E. coli* cultures were performed in 120 mL capped bottles with 50 mL of mineral medium (Diender et al., 2016). Prior to inoculation, the bottles were autoclaved at 120°C after which the mineral media was supplemented with autoclaved (120°C, 20 min); glucose 5 g L^–1^, peptone (BD Bacto Difco, Thermo Fisher Scientific, Waltham, MA, United States) 1 g L^–1^, yeast extract (BD Bacto Difco, Thermo Fisher Scientific, Waltham, MA, United States) 2 g L^–1^ and cysteine 1 g L^–1^. Additionally, 0.05 g L^–1^ ampicillin was added and the gas phase was exchanged with air, with a final pressure of 170 kPa. All *E. coli* cultures used for measurements were inoculated with overnight grown pre-cultures (1:50 v/v) and incubated at 37°C and shaken (300 rpm) until oxygen was depleted (2–3 h). Subsequently 1 mL (250 g L^–1^) glucose, 1 mL reducing agent (0.4 M cysteine) and 1 mL IPTG (40 mM) were added.

After 3 h (at 30°C, unshaken) of incubation, the cells were harvested and processed anaerobically according to Diender et al. (2016). Enzymatic activity analysis was conducted using a modified method initially described by Diender et al. (2016). The essays were performed in an anaerobic environment using 100–300 μL of cell extract with both CO and hydroxylamine as substrate. To increase metal cofactor availability, 1:200 (v/v) metals solution was added to the assay buffer which contained in (g L^–1^); HCl 1.8, H_3_BO_3_ 0.0618, MnCl_2_ 0.06125, FeCl_2_ 0.9435, CoC_l__2_ 0.0645, NiCl_2_ 0.01286, ZnCl_2_ 0.0677, CuCl_2_ 0.01335.

### Homology Protein BLAST Analysis

The sequence of the putative CODH (F7O84_RS11645) was blasted with the BLASTp (version 2.2.28+; Altschul et al., 1997) tool of the JGI-IMG/M database (Markowitz et al., 2012), with default parameter settings. Finished genomes from members of the *Lachnospiraceae* family in the public JGI-IMG/M database (Markowitz et al., 2012) were selected for analysis, Supplementary Table S4. The stains identified in the BLAST search, or closely related strains (Supplementary Table S5) were subsequently analyzed in KEGG (Kanehisa et al., 2014) for presences of the CODH/ACS complex with pathway map 1200.

## Results

### Physiological Characterization of D-Galacturonate-Limited Enrichment Cultures Dominated by “*Ca.* G. soehngenii”

Anaerobic, galacturonate-limited chemostat enrichment cultures were used to study the physiology of “*Ca.* G. soehngenii” cultures. In a previous study (Valk et al., 2018), the relative abundance of “*Ca*. G. soehngenii” in such cultures did not exceed 65%, based on metagenomic analysis, and formate and H_2_ were detected in the liquid and gas phases, respectively. It was hypothesized that, in these experiments, a low *in situ* hydrogen partial pressure limited *in vivo* WLP activity, as it was expected that hydrogen was used as reductant for the production of acetate from formate or CO_2_. To investigate this possibility, head space flushing instead of sparging was applied, using N_2_ gas. This caused an increase in the hydrogen partial pressure in the media broth (De Kok et al., 2013). Additionally, the dilution rate was decreased from 0.125 to 0.1 h^–1^. Analysis of the abundance of “*Ca*. G. soehngenii” in the resulting enrichment cultures by quantitative fluorescence *in situ* hybridization (qFISH) indicated that 86.5 ± 2.6% of the bio-volume of qFISH-detectable cells consisted of “*Ca.* G. soehngenii.” The major side population *Enterobacteriaceae* represented 13.8 ± 2.4% of the bio-volume. As these two subpopulations together accounted for 100.2 ± 5.0% of the bio-volume, it was assumed that any other, minor, subpopulations did not significantly influence the stoichiometry of catabolic fluxes.

Product yields and biomass-specific conversion rates of the D-galacturonate-limited anaerobic enrichment cultures dominated by “*Ca.* G. soehngenii” (Table 2) showed acetate as dominant catabolic product (0.57 ± 0.03 Cmol (Cmol galacturonate^–1^). Carbon and electron recoveries were 94 and 92%, respectively, indicating that all major fermentation products were identified. As observed previously (Valk et al., 2018), this acetate yield on galacturonic acid was significantly higher than the combined yields of formate and hydrogen. This difference was interpreted as indicative for acetogenesis by one of the dominant organisms, of which only the “*Ca.* G. soehngenii” MAG was shown to harbor homologs for most WLP structural genes (Ragsdale and Pierce, 2008; Valk et al., 2018). Yields of hydrogen and formate on galacturonate (0.02 ± 0.01 mol Cmol galacturonate^–1^) and 0.02 ± 0.01 (Cmol galacturonate^–1^), respectively were significantly lower than found in a previous study on “*Ca.* G. soehngenii” (Valk et al., 2018). This observation is consistent with a higher *in vivo* contribution of the WLP as a result of a higher hydrogen partial pressure and/or lower specific growth rate in the present study.

**TABLE 2:** Yields (in Cmol (Cmol galacturonate)^–1^, unless stated otherwise) and biomass- specific conversion rates (q; mmol g_*x*_^–1^ h^–1^) of anaerobic, galacturonate-limited chemostat enrichment cultures dominated by “*Ca.* Galacturonibacter soehngenii.”

	**Yield (Cmol_*i*_ Cmol_*s*_^–1^)**	**Biomass specific conversion rates (mmol (g_*x*_) ^–1^ h^–1^)**
D-galacturonate	–	−4.0 ± 0.2
Biomass	0.17 ± 0.02	–
Acetate	0.57 ± 0.03	6.9 ± 0.4
Formate	0.02 ± 0.01	0.4 ± 0.2
CO_2_	0.18 ± 0.02	4.3 ± 0.3
H_2_ (mol Cmol***^–^***^1^)	0.02 ± 0.01	0.2 ± 0.1
H_2_ + Formate (mol Cmol_*s*_***^–^***^1^)	0.04 ± 0.02	
Acetyl-CoA derivatives (mol Cmol_*s*_***^–^***^1^)	0.29 ± 0.02	

**Chemostat cultures were operated at dilution rate of 0.1 h^–1^, pH, 8 and at 30°C, with galacturonate the sole carbon-source. Data are presented as average ± mean deviations, derived from nine measurements each on duplicate steady-state enrichment cultures.**

### Incorporation of ^13^C-Labeled Bicarbonate Into Acetate Corroborates Acetogenic Fermentation

A simple model was constructed to predict formation of labeled acetate, using biomass-specific conversion rates measured in pseudo-steady state enrichment cultures as inputs (Supplementary Calculations S1, S2 and Supplementary Figure S1). Model simulations predicted that, after 8 h, approximately 15% of the acetate produced by the enrichment culture should be labeled. To investigate if CO_2_ was indeed incorporated into acetate via acetogenic fermentation, ^13^C-labeled bicarbonate was fed to a “*Ca.* G. soehngenii” enrichment chemostat culture. However, after 8 h, the fraction of ^13^C in the methyl group of acetate increased to 2.0%. This increase represented only a small increase relative to the 1% natural abundance of ^13^C (Table 3; Rumble et al., 2017). In contrast, after 8 h of ^13^C-bicarbonate feeding, the enrichment culture showed a 21.5% abundance of ^13^C in the carbonyl-group of acetate (Table 3).

**TABLE 3:** Percentages of ^13^C-labeled methyl and carbonyl groups in total-culture acetate, calculated from proton and carbon NMR spectra.

	**Time (h)**	**% ^13^C**
Methyl (CH_3_)	0	1.0
	4	1.6
	8	2.0
Carbonyl (CO)	8	21.8

**Samples were taken from the “Ca. G. soehngenii” chemostat enrichment cultures in bioreactor 2 after switching the alkali supply line from 1 M NaOH to 1 M NaH^13^CO_3_ (Time = 0 h).**

### Significant Activity of CO Dehydrogenase in Cell Extracts of “*Ca.* G. soehngenii” Enrichment Cultures

In the WLP, ^13^C-labeled CO_2_ incorporation into the carbonyl-group of acetate involves activity of CO dehydrogenase (COOS, EC 1.2.7.4). To investigate the presence of this key enzyme in “*Ca*. G. soehngenii,” an anaerobic enzyme activity assay was performed on cell extracts of enrichment cultures, using CO as electron donor and methyl viologen (MV) as electron acceptor (Diender et al., 2016). These assays revealed a CO dehydrogenase activity of 2.1 ± 0.6 μmol min^–1^ (mg protein) ^–1^. Reduction of MV in the absence of either CO or cell extract was below detection limit [<0.05 μmol min^–1^ (mg protein) ^–1^].

### Identification of Two Putative Novel CO Dehydrogenase Genes in a Newly Obtained Single-Scaffold MAG of “*Ca*. G. soehngenii”

Previous analysis of the “*Ca.* G. soehngenii” MAG (Valk et al., 2018) was based on an assembly made with short-read DNA sequencing data. To identify if putative CODH/ACS complex genes had been missed in this analysis due to incomplete assembly, long-read Oxford Nanopore sequencing (Deamer et al., 2016; Jain et al., 2016) was used to improve the previously assembled “*Ca.* G. soehngenii” MAG. The resulting genome assembly consisted of 8 contigs and was estimated to have a 98% completeness and contained no genetic contamination with sequences from other organisms according to checkM (Table 4). As in the previous study, homologs were detected for most structural genes associated with the WLP (Table 5), but none of the annotated genes in the predicted proteome showed homology with known CODH/ACS genes (Vallenet et al., 2006; Ragsdale, 2008; Valk et al., 2018). A search in the newly assembled “*Ca*. G. soehngenii” MAG sequence for homologs of signature genes of the six other known pathways for inorganic carbon fixation did not point toward their involvement in carbon metabolism (Supplementary Table S2).

**TABLE 4:** Statistics of the metagenome-assembled genome (MAG) of “*Ca.* Galacturonibacter soehngenii.”

	***“Candidatus* Galacturonibacter soehngenii”**
Genome size (Mbp)	4.1
Scaffolds	1
Contigs	8
Contigs N50	1033779
Max contig size	1514059
Completeness (%)	98
Contamination (%)	0
GC content (%)	34.4
Protein coding density (%)	89
CDS	3924
rRNA copies	5

**Completeness and contamination were estimated with CheckM (Parks et al., 2015).**

**TABLE 5:** Genes of the Wood-Ljungdahl pathway from the predictive proteome of the MAG “*Ca.* G. soehngenii” with gene names, EC number, gene or homolog and *E*-value based on SwissProt alignment (BLASTP version 2.2.28+, MicroScope platform v3.13.2).

**Encoded protein**	**EC**	**Gene name**	***E*-value**	**Gene ID**
Formate dehydrogenase	1.17.1.9	*fdhA*	1 e^–60^	F7O84_ RS07405
Formate–tetrahydrofolate ligase	6.3.4.3	*fhs*	0.0	F7O84_RS05385
Methenyl-tetrahydrofolate cyclohydrolase/methylene–tetrahydrofolate dehydrogenase	3.5.4.9 and 1.5.1.5	*folD*	5 e^–152^	F7O84_RS05380
Methyl–tetrahydrofolate reductase	1.5.1.20	*metF*	1 e^–87^	F7O84_RS08335
5-Methyl-tetrahydrofolate:corrinoid/iron-sulfur protein methyltransferase	2.1.1.258	*acsE*	5 e^–37^	F7O84_RS02745
CO-Methylating acetyl-CoA synthase	2.3.1.169	*acsBCD*	>10	
Carbon-monoxide dehydrogenase	1.2.7.4	*cooS*	>10	

CO dehydrogenases contain highly conserved amino-acid motifs (Pfam or protein-family domains) associated with their nickel-iron-sulfur clusters (Eggen et al., 1991, 1996; Maupin-Furlow and Ferry, 1996; Jeoung and Dobbek, 2011; Techtmann et al., 2012; El-Gebali et al., 2018). The newly assembled “*Ca*. G. soehngenii” MAG sequence did not reveal hits for the Pfam domain of the CO dehydrogenase α-subunit of the CODH/ACS complex (PF18537) (Darnault et al., 2003). However, two open reading frames F7O84_RS02405 and F7O84_RS11645, harbored the PF03063 Pfam domain, which is associated with the hybrid cluster protein (HCP) and the catalytic center of the Ni-CODH family (van den Berg et al., 2000; Wolfe et al., 2002). Although HCP has been associated with hydroxylamine reductase activity, its catalytic activity has not been experimentally confirmed and, moreover, sequence motifs in HCP showed high similarity with the functional domain of Ni-CODHs making it an interesting candidate genes for the CODH function of the WLP in “*Ca*. G. soehngenii” (Heo et al., 2002; Wolfe et al., 2002; Aragão et al., 2003; Almeida et al., 2006). A closer inspection of the genetic context of both genes showed many flanking genes encoding hypothetical proteins in their close vicinity, but no genes previously associated with acetogenesis.

### Homologs of Acetogenesis Genes Are Transcribed in D-Galacturonate-Limited “*Ca.* G. soehngenii” Enrichment Cultures

A meta-transcriptome analysis of the enrichment cultures showed significant transcript levels of most homologs of known WLP genes, which were approximately 10-fold lower than those of homologs of structural genes encoding Entner-Doudoroff-pathway enzymes involved in galacturonate catabolism (Table 6). A notable exception was the extremely low transcript level of a putative formate dehydrogenase gene (F7O84_RS07405; EC 1.17.1.9). A candidate gene for pyruvate-formate lyase (PFL, EC 6.2.1.3) was highly transcribed (F7O84_03160, Table 6). These observations suggested that formate generated by PFL, rather than CO_2_, was the major substrate for the methyl branch of the WLP in “*Ca*. G. soehngenii.”

**TABLE 6:** Transcript levels of putative key genes of the adapted Entner-Doudoroff pathway for galacturonate metabolism and the Wood-Ljungdahl pathway for acetogenesis in meta-transcriptome samples of the “*Ca.* G. soehngenii” chemostat enrichment cultures expressed as reads per kilobase million (RPKM, average ± average deviation) based on technical triplicates of duplicate enrichment cultures.

**Protein function**	**EC number**	**Gene ID**	**RPKM**
**Adapted entner-doudoroff pathway**			
Uronate isomerase	5.3.1.12	F7O84_RS17360	5852 ± 2398
Tagaturonate reductase	1.1.1.58	F7O84_RS17370	3067 ± 1236
Altronate dehydratase	4.2.1.7	F7O84_RS17375	8426 ± 3296
2-Dehydro-3-deoxygluconokinase	2.7.1.45	F7O84_RS17390	3863 ± 1343
2-Dehydro-3-deoxyphosphogluconate aldolase	4.1.2.14	F7O84_RS17395	1752 ± 245
**Acetate production**			
Pyruvate:ferredoxin oxidoreductase	1.2.7.1	F7O84_RS03200	4145 ± 278
Pyruvate formate lyase	6.2.1.3	F7O84_RS03160	1893 ± 651
Phosphate acetyltransferase	2.3.1.8	F7O84_RS05985	1500 ± 176
Acetate kinase	2.7.2.1	F7O84_RS05980	1625 ± 200
**Wood-Ljungdahl pathway**			
Formate dehydrogenase	1.17.1.9	F7O84_RS07405	14 ± 3
Formate–tetrahydrofolate ligase	6.3.4.3	F7O84_RS05385	256 ± 58
Methenyl-tetrahydrofolate cyclohydrolase/methylene–tetrahydrofolate dehydrogenase	3.5.4.9 and 1.5.1.5	F7O84_RS05385	236 ± 9
Methyl–tetrahydrofolate reductase	1.5.1.20	F7O84_RS08335	126 ± 13
5-methyl-tetrahydrofolate:corrinoid/iron-sulfur protein methyltransferase	2.1.1.258	F7O84_RS02745	144 ± 19
CO-methylating acetyl-CoA synthase	2.3.1.169		n.d.
CO dehydrogenase	1.2.7.4		n.d.
Prismane/CO dehydrogenase family	1.7.99.1	F7O84_RS02405	40 ± 8
Prismane/CO dehydrogenase family	1.7.99.1	F7O84_RS11645	315 ± 51
**Energy-metabolism associated genes**			
Electron transport complex protein A	7.2.1.2	F7O84_RS03295	58 ± 5
Electron transport complex protein B	7.2.1.2	F7O84_RS03300	261 ± 40
Electron transport complex protein C	7.2.1.2	F7O84_RS03275	329 ± 22
Electron transport complex protein DG	7.2.1.2	F7O84_RS03290	101 ± 13
Electron transport complex protein E	7.2.1.2	F7O84_RS03285	143 ± 9
Ferredoxin hydrogenase subunit A	1.12.7.2	F7O84_RS09545	196 ± 100
Ferredoxin hydrogenase subunit B	1.12.7.2	F7O84_RS09550	356 ± 32
Ferredoxin hydrogenase subunit C	1.12.7.2	F7O84_RS04820	124 ± 86

**N.d., not detected.**

Homologs of Rnf cluster (F7O84_03275-3295; EC 7.2.1.2) and hydrogenase (F7O84_0945-50, F7O84_04820; EC 1.12.7.2) genes, which were previously implicated in acetogenesis (Biegel and Müller, 2010; Schuchmann and Müller, 2014, 2016), showed high transcript levels (Table 6). Of the two candidate genes for CO dehydrogenase, F7O84_RS11645 showed the highest transcript level (Table 6). As, under the experimental conditions, no hydroxylamine reductase activity was expected, this result reinforced the candidature of F7O84_RS11645 as possible CO dehydrogenase gene. In an attempt to directly investigate if F7O84_RS11645 encoded a functional CO dehydrogenase, its open reading frame was cloned into high-copy-number *E. coli* expression vector. However, enzyme assays with cell extracts of the resulting *E. coli* strain did not yield consistent evidence for either CO dehydrogenase or hydroxylamine dehydrogenase activity (Supplementary Table S3).

### Identification of Proteins With a High Homology of the Putative CODH Within Other Members of the *Lachnospiraceae* Species

A protein BLAST search (Altschul et al., 1997) of the putative CODH (F7O84_RS11645) was done to investigate if presence of the putative CODH gene also coincided with an apparently incomplete WLP in other members of the *Lachnospiraceae* family. Indeed, 13 sequenced members of the *Lachnospiraceae* family showed predicted proteins with a high homology with the putative CODH (Supplementary Table S4). 9 of the 13 *Lachnospiraceae* members were present in the KEGG database (Kanehisa et al., 2014; Supplementary Table S5), and subsequently analyzed on the presence or absence of the CODH/ACS complex. All organisms contained only a partial WLP, with the ACS genes not identified. In seven of the members, respectively *Lachnoclostridium saccharolyticum*, *Lachnoclostridium phytofermentans, Pseudobutyrivibrio xylanivorans*, *Butyrivibrio fibrisolvens, Pseudobutyrivibrio xylanivorans*, and both *Roseburia* species the full CODH/ACS complex was not identified. Further study is required to elucidate the relevance of the putative CODH for acetogenic metabolism.

## Discussion

**FIGURE 1:**
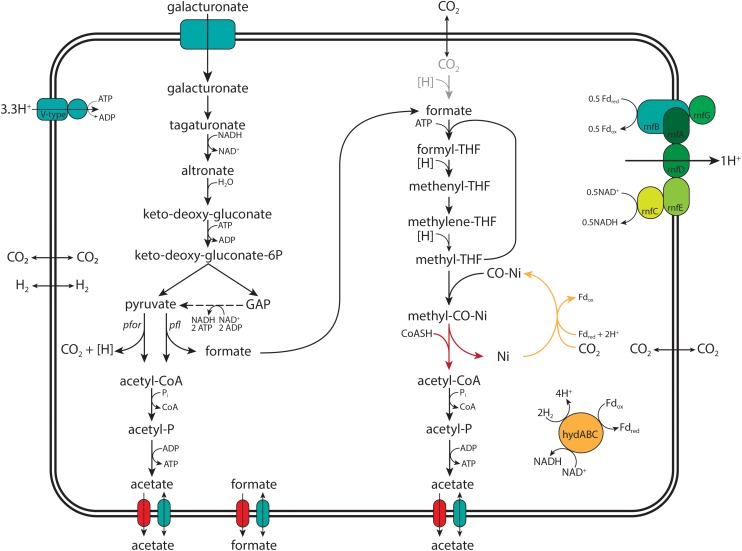
Graphical representation of the proposed pathway for acetogenic D-galacturonate catabolism in “*Candidatus* Galacturonibacter soehngenii.” The conversions of known and annotated genes identified in the MAG and transcribed in the meta-transcriptomic analysis “*Ca.* G. soehngenii” are colored black, the proposed CO dehydrogenase candidate colored yellow and the unidentified acetyl-CoA synthase colored red. With pyruvate:ferredoxin oxidoreductase (*pfor*, EC 1.2.7.1), pyruvate formate lyase (*pfl*, EC 6.2.1.3), ferredoxin hydrogenase (*hydABC*, EC 1.12.7.1) and the Rnf-cluster (*rnfABCDEG*, EC 7.2.1.2) explicitly shown.

Incorporation of carbon from ^13^C labeled bicarbonate into the carbonyl group of acetate supported our previous conclusion, based on product profiles, that acetogenesis occurs in anaerobic, galacturonate-limited enrichment culture of “*Ca.* G. soehngenii” (Valk et al., 2018). A much lower labeling of the methyl group of acetate indicated that, instead of carbon dioxide, the methyl branch of the WLP in the “*Ca.* G. soehngenii” enrichment cultures predominantly used formate as a substrate, generated in the anaerobic fermentation of galacturonate (Figure 1). This conclusion is consistent with the low transcript levels of the only putative formate dehydrogenase gene (F7O84_RS07405; EC 1.17.1.9; Table 6) identified in the “*Ca.* G. soehngenii” MAG, the high transcript level of a putative pyruvate-formate lyase gene (F7O84_RS03160, EC 6.2.1.3; Table 5) and the low net production rates of formate in the anaerobic enrichment cultures (Table 2). In contrast, previous labeling studies on acetogens harboring the WLP showed marginal preferential labeling of the carboxyl moiety of acetate (Wood and Harris, 1952; O’Brien and Ljungdahl, 1972; Schulman et al., 1972), indicating the use of extracellular CO_2_ as substrate for both the methyl- and carbonyl-groups of acetate.

While the observed labeling pattern was consistent with acetogenic metabolism of galacturonate via a WLP, this did not rule out involvement of another pathway for carbon fixation in acetate. Involvement of the hydroxypropionate bi-cycle, 3-hydroxypropionate/4-hydroxybutyrate cycle and dicarboxylate/hydroxybutyrate cycle were excluded since no homologs were found in the “*Ca.* G. soehngenii” MAG for the majority of genes associated with these three pathways (Supplementary Table S2). Key genes were also missing for the reductive pentose phosphate cycle (rPPP) and reductive citric acid cycle (rTCA) (Supplementary Table S2) and, moreover, neither of these pathways could explain preferential labeling of the carboxyl group of acetate (Alberts et al., 2002; Shimizu et al., 2015). No gene candidates were identified for the glycine cleavage (GCV) system (Supplementary Table S2 and Supplementary Figure S2) and ^13^C-labeled bicarbonate fed into this pathway should result in equal labeling of the methyl and carbonyl groups of acetate (Figueroa et al., 2018; Supplementary Figure S2). Additionally, none of the routes would require the high CO dehydrogenase enzyme activity measured in cell extracts of the “*Ca.* G. soehngenii” enrichment culture. This analysis leaves the WLP as the only known carbon fixation pathway consistent with the observed stoichiometry of fermentation products, the labeling pattern of acetate and, with the notable exception of the CODH complex, genome and transcriptome analysis of “*Ca.* G. soehngenii.”

Homologs of structural genes encoding enzymes of an adapted Entner-Doudoroff pathway for galacturonate metabolism were highly expressed in the galacturonate-limited, anaerobic “*Ca.* G. soehngenii” enrichment cultures (Table 6). Since conversion of one mole of galacturonate into two moles of pyruvate via this pathway is redox-cofactor neutral, redox equivalents for acetogenesis needed to be derived from pyruvate dissimilation (van Maris et al., 2006; Kuivanen et al., 2019). Pyruvate:ferredoxin oxidoreductase (F7O84_RS03200, EC 1.2.7.1) has been reported to couple fermentation and WLP in other anaerobes (Drake et al., 1981; Menon and Ragsdale, 1996b; Schuchmann and Müller, 2014). Strong, highly transcribed homologs of structural genes for PFOR and for a ferredoxin hydrogenase (EC 1.12.7.2) (Table 6; F7O84_RS03200 and F7O84_0945-50, F7O84_04820 respectively) indicated that it also fulfils this role in “*Ca.* G. soehngenii.”

The significant CO dehydrogenase (CODH) (Weghoff and Müller, 2016) activities in cell extracts enrichment cultures, combined with the incorporation of ^13^C from bicarbonate in acetate strongly suggested the presence of a functional CODH enzyme in “*Ca.* G. soehngenii.” Two highly conserved classes of CODH enzymes have been described (King and Weber, 2007; Techtmann et al., 2012). Aerobic CODH enzymes (*coxSML* complex; EC 1.2.5.3) have a Mo-Cu-Se associated active site and only use CO as substrate (Schübel et al., 1995; Dobbek et al., 1999). Strictly anaerobic Ni-Fe-S associated CODH (*cooS*, EC 1.2.7.4) can use also CO_2_ as substrate (Doukov et al., 2002; Ragsdale, 2008; Techtmann et al., 2012). A close functional relationship between Ni-CO dehydrogenases and hydroxylamine reductases was shown when a single amino-acid substitution was shown to change a Ni-CO dehydrogenase into a hydroxylamine reductase (Heo et al., 2002). Since no strong homologs of canonical aerobic or anaerobic CODH genes were identified, the HCP homolog F7O84_RS11645 is therefore the best candidate for the observed CODH activity. Our inability to demonstrate stable CODH activity in cell extracts upon expression of F7O84_RS11645 in *E. coli* could have many causes, including improper folding, metal or co-factor requirements (Ensign et al., 1990; Kerby et al., 1997) or requirement of additional subunits or other proteins (Bonams and Luddent, 1987; Bonam et al., 1989; Ensign and Ludden, 1991; Aragão et al., 2008; Bar-Even et al., 2012a). The immediate genetic context of F7O84_RS11645 showed many ORFs encoding predicted conserved proteins with unknown function. Co-expression of fosmid libraries (Shizuya et al., 1992; Ho et al., 2018) of the “*Ca*. G. soehngenii” MAG together with the plasmid used in this study in an *E. coli* strain, may be helpful in resolving the genetic requirements for CODH activity in this organism.

It remains unclear how the CODH-dependent carbonyl branch and formate-dependent methyl branch of a WLP pathway in “*Ca.* G. soehngenii” organism are linked. The present study is not the first in which carbon fixation linked to the WLP was observed in the absence of a full complement of canonical WLP structural genes (Zhuang et al., 2014; Figueroa et al., 2018). However, no clear physiological nor phylogenetic connections were detected between “*Ca.* G. soehngenii” and the organisms studied previously, a strict dehalogenide-respiring *Dehalococcoides mccartyi* strain from the Chloroflexi phylum and the phosphite-oxidizing Deltaproteobacterium “*Candidatus* Phosphitivorax anaerolimi” Phox-21, respectively.

This study illustrates how quantitative analysis of metabolite formation by chemostat enrichment cultures, combined with ^13^C-labeling, (meta-)genome assembly and annotation, meta-transcriptome analysis and biochemical assays can raise new and surprising questions about intensively studied metabolic pathways. Based on our results, involvement of a novel inorganic carbon assimilation pathway, which produces a similar labeling and product profile as the WLP, cannot be fully excluded. However, despite the wide distribution of the CODH/ACS complex in Bacteria and Archaea (Schuchmann and Müller, 2016), the available evidence appears to point in the direction of an as yet unidentified link between the methyl and carbonyl branches of the WLP. Further research to resolve this issue may benefit from additional labeling studies with ^13^C-bicarbonate, ^13^C-formate or partially labeled D-galacturonate combined with metabolome analysis and *in vitro* enzyme activity studies of formate dehydrogenase. Such studies are complicated by our current inability to grow “*Ca.* G. soehngenii” in pure cultures (Valk et al., 2018). The organisms shown in the Supplementary Table S4 might be interesting alternative organisms to study in more detail, as they are available in pure culture. It would therefore be relevant to identify if any of these organisms exhibit a similar acetogenic metabolism, with an incomplete complement of WLP enzymes, to further explore this intriguing metabolic conundrum.

## Data Availability Statement

The datasets generated for this study can be found in the European Nucleotide Archive (ENA) under the BioProject ID PRJNA566068, NCBI GenBank accession number MN498128.

## Author Contributions

ML, JTP, and LV designed the experiments, interpreted the results, and wrote the manuscript. LV did all cultivations and labeling study. LV and MD performed the enzyme activity assays and heterologous experiment. LV and JFP performed the qFISH analysis. GS made the model. MSD performed the experimental work for the meta-transcriptomic and meta-genomic analysis. MSD, LV, and PN analyzed the data. All authors read and approved of the final manuscript.

## Conflict of Interest

The authors declare that the research was conducted in the absence of any commercial or financial relationships that could be construed as a potential conflict of interest.

## Footnotes

1 https://www.ncbi.nlm.nih.gov/sra/

2 www.ncbi.nlm.nih.gov/genbank
