# Supplementary material for: “Candidatus Galacturonibacter soehngenii” Shows Acetogenic Catabolism of Galacturonic Acid but Lacks a Canonical Carbon Monoxide Dehydrogenase/Acetyl-CoA Synthase Complex
Source: Front Microbiol. 2020 Jan 29;11:63. doi: 10.3389/fmicb.2020.00063 (PMC7000372; doi:10.3389/fmicb.2020.00063)
Supplement: Supplementary file 2 [file Data_Sheet_2.docx]

# Supplementary Material

**Supplementary Calculations 1** – **Determination of the fraction of Wood-Ljungdahl pathway in the overall acetate production rate.**

The overall reaction of the fermentation of d-galacturonate via the adapted Entner-Doudoroff pathway is shown in equation S2.a, with formate shown as H_2_ and CO_2_ for simplification. Equation S2.b shows the overall reaction of the Wood-Ljungdahl pathway.

– 1 d-Galacturonate – 1 H_2_O + 2 H_2_ + 2 CO_2_ + 2 Acetate (S2.a)

– 4 H_2_ – 2 CO_2_ + 1 Acetate +2 H_2_O (S2.b)

Hetero-acetogenesis was hypothesized to occur in “*candidatus* Galacturonibacter soehngenii” with the total acetate production rate (mmol h^-1^) a function of the two reactions, S2.a and S2.b. The acetate production rate per pathway could be determined, with the d-galacturonate consumption rate and hydrogen and carbon dioxide production rate known, as shown in Table 1 in the main text.

The rate of S2.a could be determined by calculating the catabolic d-galacturonate consumption rate, subtracting the d-galacturonate going towards biosynthesis from the total d-galacturonate consumption rate (0.81 ± 0.01 mmol h^-1^), as d-galacturonate was solely consumed in reaction S2.a.

The total hydrogen production rate (0.18 ± 0.1 mmol h^-1^) was constituted from a hydrogen production rate (S2.a) and consumption rate (S2.b), enabling the determination of the rate of S2.b) (0.35 ± 0.03 mmol h^-1^). Based on these calculation the fraction acetate produced from the WLP was determined to be 0.35.

## Supplementary Calculations 2 – Chemostat model acetogenesis with labeled $\boldsymbol{H}{}_{\boldsymbol{6}}^{\boldsymbol{13}}\boldsymbol{C}\boldsymbol{O}_{\boldsymbol{3}}^{\boldsymbol{-}}$

A principal model was used to estimate the fraction of labeled acetate ($CH_{3}{}_{6}^{13}COOH)$ versus unlabeled acetate ($CH_{3}{}_{6}^{12}COOH)$ over time $(t)$ in a chemostat system where at $t=0$ a constant titration of $NaH{}_{6}^{13}CO_{3}$ led to a shift in the fraction of labeled versus unlabeled inorganic carbon in the reactor broth. The model assumed that the carboxyl group $(COOH)$ in the anabolism of acetate was directly derived from the dissolved inorganic carbon. Carbon dioxide transfer from the liquid to the gas phase follows the ratio of labeled versus unlabeled inorganic carbon in the liquid phase. Values for the acetate concentration, dissolved inorganic carbon concentration, carbon transfer rate, and volumetric production rates were derived from online and offline measurements (Supplementary Table S1).

Supplementary Table S1 | Kinetic and process values for chemostat model.

| **Description** | **Symbol** | **Value** | **unit** |
| --- | --- | --- | --- |
| Dilution rate | D | 0.1 | *h^-1^* |
| *CO_2_* production rate | $R_{CO_{2}}$ | 1.05 | mmol h^-1^ |
| *HCO_3_^-^* titration rate | $R_{HCO_{3}^{-}}$ | 6.0 | mmol h^-1^ |
| *Acetate* production rate | $R_{Ac}$ | 1.83 | mmol h^-1^ |
| Fraction WLP | $f_{WLP}$ | 0.35 | mmol mmol^-1^ |
| IC^a^ $(t=0, {}_{6}^{12}CO_{2})$ | $IC_{0}^{12}$ | 5 | mmol |
| IC^a^ $(t=0, {}_{6}^{13}CO_{2})$ | $IC_{0}^{13}$ | 0.05 | mmol |
| Carbon transfer rate^c^ | CTR | 2.2 | mmol h^-1^ |
| Ac^b^ $(t=0, {}_{6}^{12}CO_{2})$ | $Ac_{0}^{12}$ | 6.40 | mmol |
| Ac^b^ $(t=0, {}_{6}^{13}CO_{2})$ | $Ac_{0}^{13}$ | 0.06 | mmol |
| a. Inorganic carbon, dissolved $\boldsymbol{C}\boldsymbol{O}_{\boldsymbol{2}}$ species of labeled or unlabeled isotopes.  b. Total acetate of which the carboxyl group is either labeled or unlabeled.  c. The carbon transfer rate is the total inorganic carbon transferring from the liquid to the gas phase. | | | |

$$\begin{aligned} f_{IC^{12}}=\frac{IC^{12}}{IC^{12}+IC^{13}} \end{aligned}(1)$$

$$\begin{aligned} \frac{dIC^{12}}{dt}=-D\cdot IC^{12}+R_{CO_{2}}-f_{IC^{12}}\cdot CTR \end{aligned}(2)$$

$$\begin{aligned} \frac{dIC^{13}}{dt}=-D\cdot IC^{13}+R_{HCO_{3}^{-}}-\left( 1-f_{IC^{12}} \right)\cdot CTR \end{aligned}(3)$$

$$\begin{aligned} \frac{dAc^{12}}{dt}=-D\cdot Ac^{12}+f_{WLP}\cdot{f_{IC^{12}}\cdot R}_{Ac} \end{aligned}(4)$$

$$\begin{aligned} \frac{dAc^{13}}{dt}=-D\cdot Ac^{13}+f_{WLP}\cdot{\left( 1-f_{IC^{12}} \right)\cdot R}_{Ac} \end{aligned}(5)$$

Supplementary Figure S1 | The chemostat model of the “*ca.* G. soehngenii” enrichment culture of the expected acetate produced over time during the labelling experiment with acetate_12_ (black line (-), mmol) and acetate_13_ (dark grey line (-), mmol), the total amount of acetate produced (light grey line (-), mmol) from the Wood-Ljungdahl pathway, the percentage of labelled acetate versus unlabeled acetate in the reactor broth (dotted grey line
(- -), %) and the labelled acetate measured after 8 h (×, %) in bioreactor 2.

Supplementary Table S2 | Genes of the six carbon fixation pathways, excluding the Wood-Ljungdahl pathway, from the predictive proteome of the MAG “*ca*. G. soehngenii” with gene ID and EC number based on SwissProt alignment (BLASTP version 2.2.28+, MicroScope platform v3.13.2).

| **Route** | **EC number** | **Gene ID** |
| --- | --- | --- |
| ***Reductive pentose phosphate cycle (CBB)*** |  |  |
| Ribulose-bisphosphate carboxylase | 4.1.1.39 |  |
| Phosphoglycerate kinase | 2.7.2.3 | F7O84_RS02895 |
| Glyceraldehyde-3-phosphate dehydrogenase | 1.2.1.12 | F7O84_RS02890 |
| Triose phosphate isomerase | 5.3.1.1 | F7O84_RS02900 |
| Fructose-1,6-bisphosphate aldolase | 4.1.2.13 | F7O84_RS12150 |
| Fructose-1,6-bisphosphatase class 3 | 3.1.3.11 | F7O84_RS00620 |
| Transketolase | 2.2.1.1 | F7O84_RS11220-25 |
| Sedoheptulose-bisphosphatase | 3.1.3.37 |  |
| Ribose-5-phosphate isomerase A | 5.3.1.6 | F7O84_RS11205 |
| Ribulose-phosphate 3-epimerase | 5.1.3.1 | F7O84_RS11250 |
| ***Reductive citric acid cycle*** |  |  |
| Pyruvate ferredoxin:oxidoreductase | 1.2.7.1 | F7O84_RS03200 |
| Aconitate hydratase | 4.2.1.3 | F7O84_RS03605 |
| 2-oxoglutarate/2-oxoacid ferredoxin oxidoreductase | 1.2.7.3 and 1.2.7.11 | F7O84_RS03615-20 |
| Malate dehydrogenase | 1.1.1.37 | F7O84_RS05485 |
| Isocitrate dehydrogenase (NADP+) | 1.1.1.42 | F7O84_RS06610 |
| Propionyl-CoA carboxylase | 6.4.1.3 | F7O84_RS07040 |
| Pyruvate phosphate dikinase | 2.7.9.1 | F7O84_RS07755 |
| Pyruvate carboxylase subunit B | 6.4.1.1 | F7O84_RS12740 |
| Aconitate hydratase | 4.2.1.3 | F7O84_RS14165 |
| Fumarate hydratase | 4.2.1.2 | F7O84_RS14815 |
| ATP citrate lyase | 2.3.3.8 |  |
| Phosphoenolpyruvate carboxylase | 4.1.1.31 |  |
| Fumarate reductase | 1.3.5.4 |  |
| Succinate-CoA ligase | 6.2.1.5 |  |
| ***Hydroxypropionate bi-cycle*** |  |  |
| Pyruvate ferredoxin:oxidoreductase | 1.2.7.1 | F7O84_RS03200 |
| pyruvate phosphate dikinase | 2.7.9.1 | F7O84_RS07755 |
| Phosphoenolpyruvate carboxylase | 4.1.1.31 |  |
| Malate dehydrogenase | 1.1.1.37 | F7O84_RS05485 |
| Fumarate hydratase | 4.2.1.2 | F7O84_RS14815 |
| Succinate dehydrogenase | 1.3.5.1 |  |
| Fumarate reductase | 1.3.4.1 |  |
| Fumarate reductase (NADH) | 1.3.1.6 |  |
| Fumarate reductase (quinol) | 1.3.5.4 |  |
| Succinate-CoA ligase | 6.2.1.5 |  |
| ***3-Hydroxypropionate/4-Hydroxybutyrate cycle*** |  |  |
| Acetyl-CoA carboxylase | 6.4.1.2 |  |
| Manonyl-CoA reductase (NADP dependent) | 1.2.1.75 |  |
| 3-hydroxypropionate dehydrogenase (NADP+) | 1.1.1.298 |  |
| 3-hydroxypropionyl-CoA synthase | 6.2.1.36 |  |

Table S2 | *Continued*

| 3-hydroxypropionyl-CoA dehydratase | 4.2.1.116 |  |
| --- | --- | --- |
| Acrylyl-CoA reductase (NADPH) | 1.3.1.84 |  |
| Propionyl-CoA carboxylase | 6.4.1.3 | F7O84_RS03215 |
| Methylmalonyl-CoA epimerase | 5.1.99.1 |  |
| Methylmalonyl-CoA mutase | 5.4.99.2 |  |
| Succinyl-coA reductase | 1.2.1.76 |  |
| Succinate semialdehyde reductase (NADPH) | 1.1.1.- |  |
| 4-hydroxybutyrate-CoA ligase | 6.2.1.40 |  |
| 4-hydroxybutanoyl-CoA dehydratase | 4.2.1.120 |  |
| Enoyl-CoA hydratase | 4.2.1.17 |  |
| 3-hydroxyacyl-CoA dehydrogenase | 1.1.1.35 |  |
| Acetyl-CoA acetyltransferase | 2.3.1.9 |  |
| Malyl-CoA lyase | 4.1.3.24 |  |
| 2-methylfumaryl-CoA hydratase | 4.2.1.148 |  |
| 2-methylfumaryl-CoA isomerase | 5.4.1.3 |  |
| 3-methylfumaryl-CoA hydratase | 4.2.1.153 |  |
| (S)-citramalyl-CoA lyase | 4.1.3.25 |  |
| ***Dicarboxylate/hydroxybutyrate cycle*** |  |  |
| Malyl-CoA lyase | 4.1.3.24 |  |
| Succinyl-Coa l-malate-CoA transferase | 2.8.3.22 |  |
| Malate dehydrogenase | 1.1.1.37 | F7O84_RS05485 |
| Phosphoenolpyruvate carboxylase | 4.1.1.31 |  |
| Pyruvate phosphate dikinase | 2.7.9.1 | F7O84_RS07755 |
| Pyruvate:ferredoxin oxidoreductase | 1.2.7.1 | F7O84_RS03200 |
| ***Reductive glycine pathway*** |  |  |
| Formate dehydrogenase | 1.17.1.9 | F7O84_RS07405 |
| Ferredoxin hydrogenase | 1.12.7.2 | F7O84_RS09545-50 F7O84_RS04820 |
| Formate-tetrahydrofolate ligase | 6.3.4.3 | F7O84_RS05385 |
| Methylenetetrahydrofolate dehydrogenase | 3.5.4.9 | F7O84_RS05380 |
| Glycine cleavage system H protein |  |  |
| Dihydrolipoamide dehydrogenase | 1.8.1.4 |  |
| Glycine cleavage system T protein | 2.1.2.10 |  |
| Glycine dehydrogenase subunit A | 1.4.4.2 |  |
| Glycine dehydrogenase subunit B | 1.4.4.2 |  |
| Glycine hydroxymethyl transferase | 2.1.2.1 | F7O84_RS01545 |
| Serine dehydratase | 4.3.1.17 | F7O84_RS04460-5 |

**Supplementary Table S3 |** Enzyme assay of the putative CO dehydrogenase expressed in an *E. coli* BL21 strain. The mean ± average deviation have been determined from independent duplicate experiments. B.d.l. below detection limit.

| CO | Hydroxylamine | Metals | pUD1074 | *E. coli* BL21 | Activity (slope/min) |
| --- | --- | --- | --- | --- | --- |
| + | - | + | + | - | 0.013 ± 0.013 |
| - | + | + | + | - | 0.02 |
| + | - | - | + | - | b.d.l. |
| - | - | + | + | - | b.d.l. |
| + | - | + | - | + | b.d.l. |
| - | + | + | - | + | b.d.l. |
| + | - | + | - | - | b.d.l. |

Supplementary Table S4 | BLASTp (version 2.2.28+, (Altschul et al., 1997)) search with the organism, genome id, gene (locus tag) and E-value, of the putative CODH (F7O84_RS11645) in *Lachnospiraceae* species obtained from the public JGI-IMG/M database (Markowitz et al, 2012).

| Strain | Genome id | Gene | E-value |
| --- | --- | --- | --- |
| *Anaerobutyricum* *hallii* L2-7 | 2826986812 | Ga0265454_2814 | 0 |
| *Pseudobutyrivibrio xylanivorans* MA3014 v2 | 2836274772 | Ga0400149_2313 | 0 |
| *Butyrivibrio fibrisolvens* INBov1 | 2841549333 | Ga0364991_2769 | 0 |
| *Lachnoclostridium phytofermentans* ISDg | 641228486 | Cphy_2846 | 0 |
| *Lachnoclostridium saccharolyticum* WM1, DSM 2544 | 648028018 | Closa_2590 | 0 |
| *Lachnoclostridium* cf. *saccharolyticum* K10 | 650377923 | CLS_14500 | 0 |
| *Roseburia hominis* A2-183, DSM 16839 | 2511231128 | RHOM_10215 | 7.0 e^-178^ |
| *Roseburia intestinalis* L1-82 | 2836830276 | Ga0399605_1213 | 1.0 e^-175^ |
| *Roseburia intestinalis* M50/1 | 650377964 | ROI_37590 | 2.0 e^-175^ |
| *Roseburia intestinalis* XB6B4 | 650377965 | RO1_26150 | 2.0 e^-175^ |
| *Anaerobutyricum hallii* L2-7 | 2826986812 | Ga0265454_1517 | 3.0 e^-173^ |
| *Roseburia hominis* A2-183, DSM 16839 | 2511231128 | RHOM_06150 | 1.0 e^-166^ |
| *Lachnoclostridium saccharolyticum* WM1, DSM 2544 | 648028018 | Closa_2549 | 6.0 e^-165^ |
| *Roseburia intestinalis* L1-82 | 2836830276 | Ga0399605_896 | 8.0 e^-160^ |
| *Roseburia intestinalis* XB6B4 | 650377965 | RO1_03190 | 1.0 e^-159^ |
| *Roseburia intestinalis* M50/1 | 650377964 | ROI_29330 | 2.0 e^-159^ |
| *Lachnoclostridium* sp. YL32 | 2721755796 | Ga0175994_113462 | 4.0 e^-152^ |
| *Lachnoclostridium bolteae* ATCC BAA-613 | 2825694722 | Ga0225995_2847 | 3.0 e^-150^ |
| *Lachnoclostridium phytofermentans* ISDg | 641228486 | Cphy_2847 | 2.0 e^-134^ |

Table S5 | Strain, KEGG T number and pathway map used for the KEGG analysis (Kanehisa et al., 2014) for the presence of the CODH/ACS complex.

| Strain | KEGG T number | Pathway map |
| --- | --- | --- |
| *Anaerobutyricum hallii* EH1 | T05310 | ehl01200 |
| *Pseudobutyrivibrio xylanivorans* MA3014 | T06217 | pxv01200 |
| *Butyrivibrio fibrisolvens* 16/4 | T02581 | bfi01200 |
| *Lachnoclostridium phytofermentans* ISDg | T00619 | cpy01200 |
| *Clostridium saccharolyticum* WM1, DSM 2544 | T01288 | csh01200 |
| *Clostridium* cf. *saccharolyticum* K10 | T02614 | cso01200 |
| *Roseburia hominis* A2-183, DSM 16839 | T01621 | rho01200 |
| *Roseburia intestinalis* M50/1 | T02598 | rim01200 |
| *Roseburia intestinalis* XB6B4 | T02597 | rix01200 |
| *Lachnoclostridium* sp. YL32 | T04430 | lacy01200 |
| *Clostridium bolteae* ATCC BAA-613 | T05466 | cbol01200 |


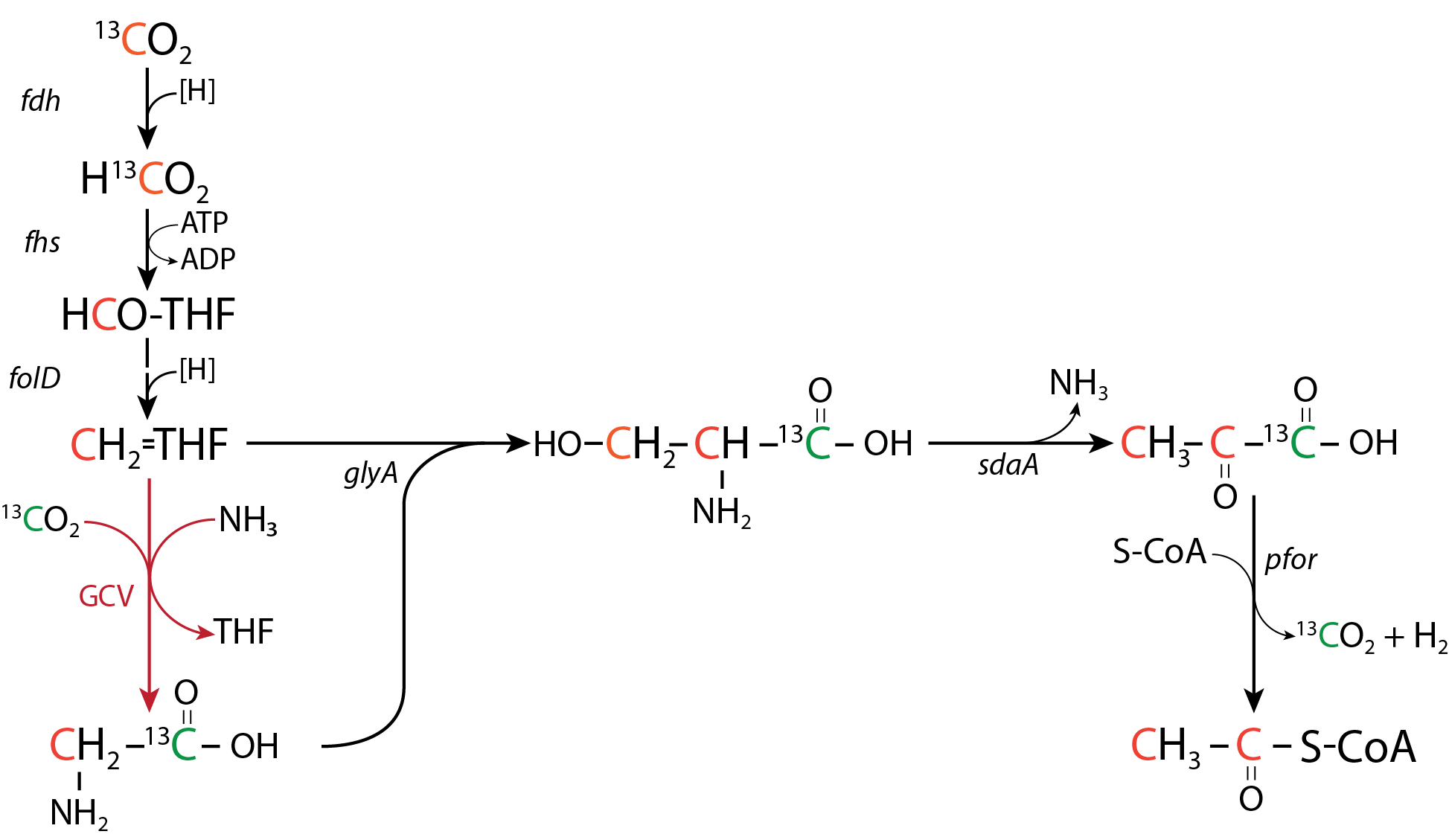


Supplementary Figure S2 | The predicted ^13^C-labelling pattern produced by the reductive glycine pathway, with formate dehydrogenase *fdh*, formate-tetrahydrofolate ligase *fhs*, methylenetetrahydrofolate dehydrogenase *folD*, GCV (glycine cleavage system (GcvH, lipoate-binding protein; GcvP, glycine dehydrogenase; GcvT, aminomethyltransferase; Lpd, dihydrolipoyl dehydrogenase); *glyA*, serine hydroxymethyltransferase; *sdaA*, serine deaminase and *pfor*; pyruvate ferredoxin oxidoreductase. The GCV depicted in red was not identified in the genome of “*ca.* G. soehngenii” (Supplemental material Table S2), the ^13^C-labelled carbon integrated via the methyl-branch is depicted in orange and the ^13^C-labelled carbon integrated via the GCV is depicted in green. (*adapted from* (Figueroa et al., 2018)).
